# Polar/Ionizable Residues in Transmembrane Segments: Effects on Helix-Helix Packing

**DOI:** 10.1371/journal.pone.0044263

**Published:** 2012-09-12

**Authors:** Manuel Bañó-Polo, Carlos Baeza-Delgado, Mar Orzáez, Marc A. Marti-Renom, Concepción Abad, Ismael Mingarro

**Affiliations:** 1 Departament de Bioquímica i Biologia Molecular, Universitat de València, Burjassot, Spain; 2 Centro de Investigación Príncipe Felipe, Valencia, Spain; 3 Genome Biology Group, Structural Genomics Team, Centre Nacional d'Anàlisi Genòmic, Barcelona, Spain; 4 Structural Genomics Group, Center for Genomic Regulation, Barcelona, Spain; Nagoya University, Japan

## Abstract

The vast majority of membrane proteins are anchored to biological membranes through hydrophobic α-helices. Sequence analysis of high-resolution membrane protein structures show that ionizable amino acid residues are present in transmembrane (TM) helices, often with a functional and/or structural role. Here, using as scaffold the hydrophobic TM domain of the model membrane protein glycophorin A (GpA), we address the consequences of replacing specific residues by ionizable amino acids on TM helix insertion and packing, both in detergent micelles and in biological membranes. Our findings demonstrate that ionizable residues are stably inserted in hydrophobic environments, and tolerated in the dimerization process when oriented toward the lipid face, emphasizing the complexity of protein-lipid interactions in biological membranes.

## Introduction

The vast majority of membrane proteins are anchored to biological membranes through hydrophobic α-helices. These transmembrane (TM) α-helices, rather than serving solely as featureless hydrophobic stretches required for anchorage of proteins in membranes, have structural and/or functional roles well beyond this canonical capacity. In fact, the folding and assembly of membrane proteins rely in part on interacting TM helices, which was conceptualized as a two-stage process [1]. In the first stage, TM helices are inserted into the membrane by the translocon. The driving force for this process derives primarily from the transfer of hydrophobic side chains from the aqueous channel of the translocon to the apolar region of the bilayer [2]. In the second stage, the protein attains its native tertiary structure through the packing of its TM helices. In the apolar environment of the membrane core, van der Waals packing, hydrogen bonding and ionic interactions are the dominant contributors to TM helix packing.

Sequence analysis of high-resolution membrane protein structures show that ionizable amino acid residues are present in TM helices, although at a low frequency level [3]. Insertion of these residues through the translocon has been proved to be feasible thanks to the overall hydrophobicity of the TM segment [4] and depending on their position along the hydrophobic region [5]. In many cases, ionizable residues are involved in TM helix packing [6], [7], [8]. Likely, hydrogen bonding [6], [7] or salt-bridge [9] formation with other membrane-spanning hydrophilic residues drives these interactions, while at the same time, reduces the unfavorable energetics of inserting polar or ionizable residues into the hydrophobic membrane core.

Homo-oligomeric membrane proteins provide attractive systems for the study of TM helix packing because of their symmetry and relative simplicity. These model systems can serve as an excellent starting point to understand the structural dynamics and folding pathways of larger membrane proteins. One of the best-suited models of membrane protein that oligomerizes (more specifically, dimerizes) through non-covalent interactions of its TM α-helix is undoubtedly glycophorin A (GpA) [10], [11]. The wide use of this protein as a model membrane protein is partially based on its intrinsic simplicity, since the free energy decrease associated with TM helix-helix interactions is enough to confer detergent resistant dimerization to the protein. Thus, those factors that could affect or modify the dimerization process can be analyzed using sodium dodecyl sulfate (SDS)-PAGE. The GpA homodimer, defines a dimerization interface that has been extensively studied by diverse techniques such as saturation mutagenesis [12] and alanine-insertion scanning [13] in SDS micelles, solution NMR in dodecyl phosphocholine micelles [14] and solid-state NMR in lipid membranes [15]. The output of these studies describes a dimerization motif in the TM segment composed of seven residues, L^75^IxxGVxxGVxxT^87^, which is responsible for the dimerization process. More recently, using proline-scanning mutagenesis it was demonstrated that Leu75 is not so cleanly involved in the packing process [16], focusing the interaction on the central G^79^VxxGVxxT^87^ motif, which includes the widely proved framework for TM helix association, GxxxG [17], [18]. Nevertheless, the sequence context highly determines the thermodynamic stability of GxxxG-mediated TM helix-helix interactions (recently reviewed [19]).

In the present study, we have analyzed the distribution of ionizable (Asp, Glu, Lys and Arg) amino acid residues in TM segments from high-resolution membrane protein structures, which have to energetically accommodate into the highly hydrophobic core of biological membranes by interacting favorably with its local environment. Then, we address the consequences of replacing specific residues by ionizable amino acids along the hydrophobic region of the GpA TM domain on the dimerization of this model membrane protein, both in detergent micelles and in biological membranes. Our findings demonstrate that ionizable residues are stably inserted in hydrophobic environments, and tolerated in the dimerization process when oriented toward the lipid face, emphasizing the complexity of protein-lipids interactions in biological membranes.

## Results and Discussion

### Ionizable amino acid residues in TM α-helices

TM helices of lengths between 17 and 38 residues were selected from the MPTOPO database [20], which included helical segments that do completely span the hydrophobic core of the membrane. TM helices shorter than 17 residues as well as larger than 38 residues were excluded since they may not cross entirely the membrane or may contain segments parallel to the membrane [3], respectively.

**Figure 1:**
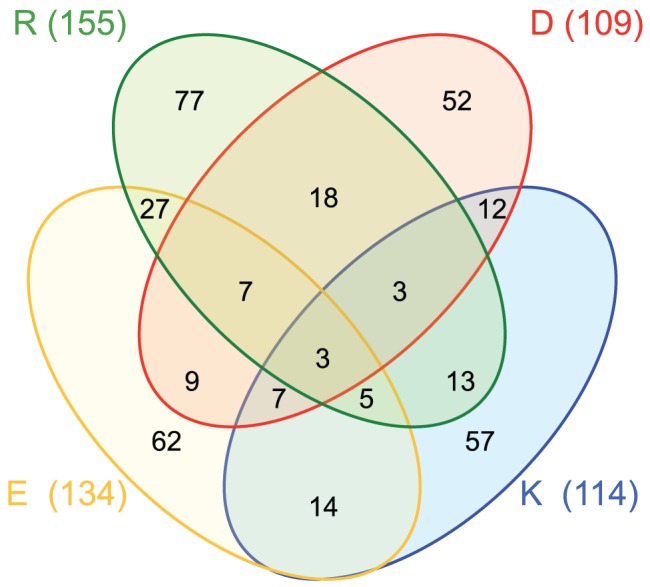
Venn diagram of TM segments (the central 19 residues) containing charged residues: Asp (D, red), Lys (K, blue), Glu (E, yellow) and Arg (R, green). The value in parenthesis is the total TM helices that contain at least one of such residues. The values inside the ellipses indicate the number of TM helices in each combination of these four amino acids. For example, there are 57 TM helices with only Lys as a charged residue, 12 helices with only Lys and Asp, 7 helices with Lys, Asp and Glu, and 3 helices with all four ionizable residues.

**Figure 2:**
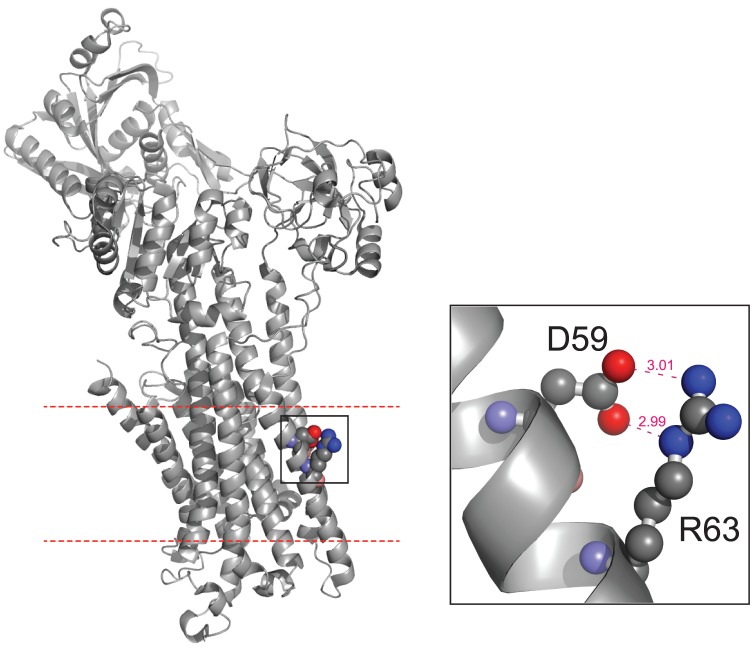
Structure of the calcium ATPase 1. **Left panel:** cartoon representation of sarcoplasmic/endoplasmic reticulum calcium ATPase 1 (PDB ID: 1SU4) with cytosolic domain in the up side and transmembrane aspartic 59 and arginine 63 residues in spheres representation (C atom gray, O atom red and N atom blue). Membrane boundaries (dashed red lines) were obtained from the *PPM Server* [44]. **Right panel:** zoom view centered on the salt bridge between Asp59 and Arg63, dashed pink lines indicate O to N atom distances.

As expected, ionizable residues (Asp, Glu, Lys, and Arg) are present at a low frequency level. All together, these residues constitute only 6.6% of the residues within TM helices. Despite their lower presence, strongly polar residues are evolutionary conserved in TM proteins, which can be partially explained by their tendency to be buried in the protein interior and also in many cases due to their direct involvement in the function of the protein [21], [22]. Among the 792 TM helices included in our database, 366 helices (46.2%) contained at least one ionizable residue within the hydrophobic region (that is, the central 19 amino acid residues). A summary of the statistics is presented in Figure 1. Furthermore, 96 TM helices contained at least one acidic plus one basic residue in their sequence, and 20 of these helices present oppositely charged residues with the appropriate periodicity (*i*, *i*+4) to form intrahelical charge pairs. To gain more detailed insight into the structural role of these ionizable residues within the membrane core, we analyzed the environment of all these 20 helices. Approximately half of the ionizable residues (51%) found in these helices are buried in the protein interior, but the rest are partly exposed to the lipid face. Some of these lipid facing ionizable residues are located in pairs at the appropriate distance to form a salt-bridge, as in the sarcoplasmic/endoplasmic reticulum calcium ATPase 1 protein (Fig. 2).

### Effects on SDS-resistant TM helix packing

**Figure 3:**
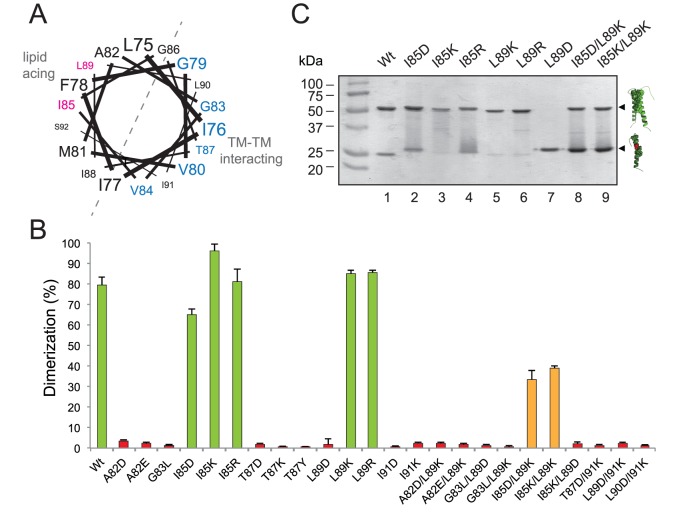
Dimerization in SDS micelles. (A) Helical wheel projection of GpA TM sequence. The residues associated with dimer formation as defined by Orzaez et al. [16] are shown in blue. Non-interacting residues susceptible of ionizable residue substitution are shown in magenta. (B) Green colored bars denoted dimerization levels similar to wild-type sequence. Bars for intermediate dimerization levels (≈40%) are colored orange. Red colored bars denote non-dimerizing sequences (dimerization<3%). (C) SDS-PAGE analysis of GpA mutants. Chimeric proteins were purified in the presence of SDS and analyzed by PAGE. Positions of the monomer and dimer of the chimeras are marked on the right as single and double helices, respectively.

Next, we investigated the effect of strongly polar residues in TM helix packing using the GpA TM segment as a model (scaffold) segment. Initial polar mutations (T87D, T87K, I91D, and I91K) made on residues located at the helix-helix interface (Fig. 3A) abolished dimerization (Fig. 3B). Furthermore, it has been reported that T87S (which retains the side chain γ oxygen) permits dimer formation both in SDS micelles [23] and in *E. coli* membranes [24], whereas a bulkier hydroxylated side chain (T87Y) is strongly disruptive (Fig. 3B). However, point mutations corresponding to replacements of nonpolar residues located at the lipid-facing interface (Fig. 3A) by ionizable residues gave rise to a more tolerated response (Fig. 3B). When Ile85 was substituted by ionizable side-chain residues, either negatively charged (I85D) or positively charged (I85K and I85R), the dimerization level was similar to native GpA TM sequence as shown under SDS-PAGE analysis (Fig. 3C, compare lanes 2, 3 and 4 to lane 1). It is commonly assumed that single ionizable residues should exist in their uncharged form within membrane-spanning helices [25]. In fact, the *pK*_a_ values observed for Asp residues in hydrophobic helices were somewhat elevated (5–8.5) relative to those for Asp residues in solution [26]. Furthermore, the replacement of Leu89 by basic residues (L89K and L89R) had almost no effect, while its substitution by an acidic residue (L89D) abolished dimerization (Fig. 3B and 3C). The opposing consequences observed for Leu89 mutants can be explained taking into account the nature of the SDS-micelles used in these experimental conditions. These results suggest that L89D mutation alters the interaction of the protein with the negatively charged detergent micelle, possibly resulting in a structure that differs from a ‘transmicellar’ α-helix due to helix distortions and interaction with the polar micelle surface. This effect was not observed when the Asp residue was located in a more central position (I85D), where its carboxylate should be located away from the negatively charged sulfate groups of the SDS molecules. In this regard, the capacity of SDS to respond to such nuance of sequence in terms of SDS solvation of TM segments within protein-SDS detergent complexes has been proved to be highly sequence (position) dependent [27]. Nevertheless, the comparable electrophoretic migration observed for I85D and L89D (Fig. 3C) suggests that the monomers associate with SDS quite similarly. To identify the helix interface responsible of dimer formation in the Leu89 mutants, we designed double mutants that contained a non-polar highly disruptive mutation (G83L). Gly83 has been proved to be extremely sensitive, since all mutations tested disrupted the dimer completely [12]. As shown in Fig. 3B, G83L mutant did not form any detectable dimer, and both double mutant proteins (G83L/L89K and G83L/L89D) containing this mutation did not dimerized, suggesting that the lysine residue introduced was not participating in the dimerization process, instead, the native dimerization motif is responsible of helix-helix interaction.

Given the 3.6-residue periodicity of an ideal α-helix, intrahelical charge pairs would be expected for (*i*, *i*+4) Lys-Asp pairs. To further assess if intrahelical charge pair formation can be tolerated in dimerizing TM sequences, we performed a double mutation combining two strongly dimerizing sequences (I85D/L89K), which only reduced dimerization by about 50% compared to the wild-type sequence (Fig. 3B). Similarly, I85K/L89K mutant retained the same level of dimerization, likely favored by a beneficial SDS solvation effect on the lysine residues. On the contrary, when oppositely charged residues were located at the TM-interacting interface (T87D/I91K) dimerization was abrogated (Fig. 3B). Furthermore, when charge pairs include L89D mutation although facing the lipids, as for I85K/L89D, we found no evidence for dimer formation (Fig. 3B). These results suggest that charge pairs are tolerated only when located at the non-interacting interface, but solely at specific positions.

Recent mutational analysis of strongly self-interacting TM segments demonstrated that basic and acidic residues located at the helix-interacting interface participate in homotypic interactions [25]. In this case, basic and acidic residues spaced (*i*, *i*+1) and (*i*, *i*+2) contribute to the interaction of model TM segments. To test this idea in the GpA sequence, we designed two mutants with appropriately spaced basic and acidic residues (L89D/I91K and L90D/I91K), and no dimeric forms were observed in any of these proteins.

In light of our experiments in SDS micelles, it can be concluded that nonpolar to ionizable substitutions away from the dimer interface (lipid facing) in combination with N-terminal native GpA dimerization motif (including GxxxG sequence) does not perturb the dimerization process, while similar mutations positioned at the helix-interacting interface strongly compromise dimer formation.

### Effects on insertion and packing into biological membranes

**Figure 4:**
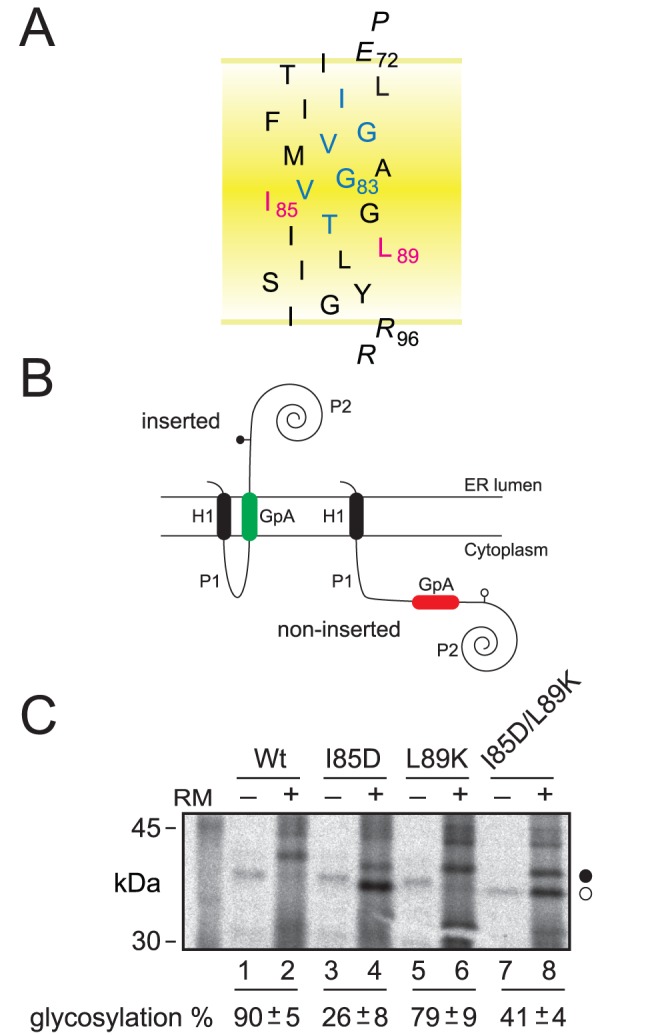
Insertion of GpA-derived segments into microsomal membranes. (A) Model of the GpA TM wild-type sequence. GpA residues involved in dimer formation are blue, the hydrophobic residues replaced to ionizable residues are magenta, and flanking residues are shown in italic. (B) Membrane topology of Lep chimeras. The second TM segment of Lep was replaced by the GpA TM amino acid sequence (gray). The glycosylation acceptor site located in the beginning of the P2 domain will be modified only if GpA-derived TM sequence inserts into the membrane. (C) *In vitro* translation in the presence of rough microsomal membranes (RM). Proper insertion of the GpA-derived TM sequences results in an increase in molecular mass of about 2.5 kDa relative to the observed molecular mass of the proteins expressed in the absence of microsomes. Bands of nonglycosylated and glycosylated proteins are indicated by white and black dots, respectively. Average ± s.d. of glycosylation results from four independent experiments are shown at the bottom.

To test the molecular effect of the ionizable residues in biological membranes we used a glycosylation mapping technique to measure changes in the insertion capacity of the GpA TM domain after introduction of ionizable residues at the more tolerant positions in terms of TM packing. The glycosylation mapping technique has been used previously to investigate the membrane insertion level of hydrophobic regions and to systematically examine the effects of individual residues on their position in the membrane [16], [28], [29]. The method is based on the observation that the endoplasmic reticulum (ER) enzyme oligosaccharide transferase (OST) can only transfer a sugar moiety to Asn-X-Thr/Ser acceptor sites when they are oriented toward the lumen of the ER membrane. To assess the effect of the presence of ionizable residues on the GpA TM segment insertion into biological membranes, we located this hydrophobic sequence (Fig. 4A) in place of the second TM fragment of the well-characterized *Escherichia coli* inner membrane protein leader peptidase (Lep). Although of bacterial origin, Lep integrates efficiently into dog pancreas microsomes with the same topology as in *E. coli* [30] (*i.e.*, with both the N- and C-termini exposed to the luminal side of the ER membrane) and the presence of its first TM segment together with the cytoplasmic P1 domain (Figure 4B) is sufficient for proper targeting of chimeric proteins to the eukaryotic membrane [30], [31]. An engineered glycosylation site placed at the C-terminal P2 domain is glycosylated efficiently upon correct insertion into the microsomal membrane (Fig. 4B), serving as a reporter to distinguish between a lumenal (glycosylated) and a cytoplasmic (unglycosylated) location. Glycosylation of the molecule results in an increase in molecular mass of about 2.5 kDa relative to the observed molecular mass of Lep expressed in the absence of microsomes. The efficiency of glycosylation of Lep under standard conditions is 80–90% [31], [32]. The strength of the Lep system is that it provides a comparative scale for the energetic cost of inserting a broad range of model and actual TM sequences into biological membranes, closely mimicking the *in vivo* situation.

The wild-type sequence of GpA TM segment efficiently inserts into the ER-derived microsomal membranes, while I85D mutation severely diminished membrane insertion capacity (Fig. 4C). On the contrary, L89K mutation allowed efficient insertion (Fig. 4C, lane 6). The different effect observed for these two mutants can be explained by differences in amino acid side chain size and the position of the residue in relation to the midpoint of the TM sequence (Fig. 4A). Hence, in the case of L89K, the longer side chain of this cationic amino acid and its proximity to the membrane interface compared to I85D may allow the hydrophilic moiety of the lysine residue to snorkel, that is, to approach its ε-amino group toward the interfacial and aqueous region, close to the negatively charged phospholipid head groups. Next, a construct with an Asp-Lys pair at the same positions (double mutant I85D/L89K) was glycosylated somewhat more efficiently than the I85D construct (Fig. 4C, lanes 4 and 8), supporting the idea that an intrahelical salt-bridge or hydrogen bond interactions between Lys and Asp side chains located on the same face of a TM helix can facilitate its insertion into biological membranes by reducing the free energy of membrane partitioning, as previously suggested in a similar system [9]. Furthermore, the predicted insertion frequencies from the biological hydrophobicity scale [2], [5] for these mutants using the ΔG Prediction Server v1.0 (http://dgpred.cbr.su.se/) are shown in Table 1. In this algorithm, the predicted insertion frequency comes from the apparent free-energy difference (ΔG_app_) from insertion into ER membranes. Since very low and very high insertion efficiencies cannot be accurately measured, ΔG_app_ values outside the interval ±1.5 kcal/mol are only qualitative. The positive value of ΔG_app_ predicted that the tested-sequence is not TM. The high negative value for the GpA wild-type sequence agrees with our experimentally measured glycosylation data showing the highest insertion efficiency. A closer analysis of the output data highlighted I85D mutation as precluding TM disposition. Hence, replacing Ile85 with aspartic acid reduced ΔG_app_ by almost 2 kcal/mol (Table 1), which correlates with our lowest glycosylation efficiency. However, replacing Leu89 with lysine has a lower energy cost (ΔG_app_ close to 0) that is reflected by a higher insertion level (Fig. 4C). Finally, the double mutant I85D/L89K results in the highest predicted penalty for TM disposition, whereas experimentally we find no evidence that GpA TM segment is significantly compromised by the presence of two poplar/ionizable residues. Such phenomena points towards an intra-helical interaction between the ionizable residues and should be taken into account to improve TM prediction algorithms.

**Table 1:** Thermodynamic cost of GpA-derived TM segments integration.

GpA-derived region	ΔG_pred_	Glycosylation % (measured)	Sequence
Wt	−1.646	90±5	ITLIIFGVMAGVIGTILLISYGI
I85D	+ 0.413	26±8	ITLIIFGVMAGV**D**GTILLISYGI
L89K	+ 0.112	79±9	ITLIIFGVMAGVIGTI**K**LISYGI
I85D/L89K	+ 2.561	41±4	ITLIIFGVMAGV**D**GTI**K**LISYGI

The predicted (ΔG_pred_) energetic cost in kcal/mol of inserting versions of the GpA TM spanning region estimated using the biological hydrophobicity scale [2], [5] are provided solely for the basis of comparison. Negative ΔG_pred_ values are indicative of TM disposition, while positive values indicate non-TM disposition. Mutated residues at positions 85 and 89 are shown in bold.

**Figure 5:**
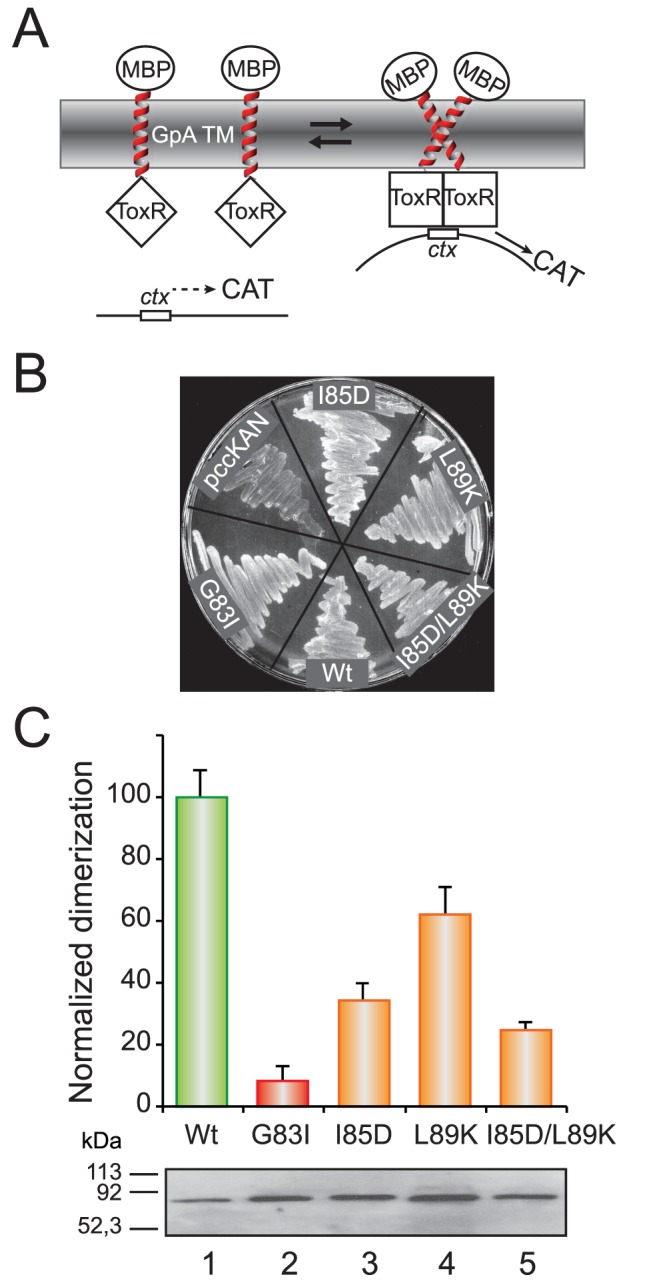
Dimerization in *E. coli* membranes. (A) Schematic representation of the ToxCAT assay. ToxR domains (squares) can activate transcription of the reporter gene (CAT) if brought together by the GpA-derived TM domains (right). The maltose binding protein domain (ellipses) helps direct the insertion of the construct into the membrane, complements the *malE* mutation in the host cells, and serves as an epitope for quantifying the expression level of fusion protein. (B) Complementation assays for wild-type and selected mutant ToxR(GpA)MBP fusion constructs. NT326 cells (*malE* deficient) carrying various constructs were streaked on a plate with maltose as the sole carbon source and grown for three days at 37°C. All ToxR(GpA)MBP chimeras permit growth of NT326 cells on maltose, while control transformants (pccKAN) do not. (C) Normalized dimerization of the indicated TM domain variants as measured by CAT-ELISA relative to the wild-type GpA TM domain. Bars for intermediate dimerization and non-dimerizing levels are colored orange and red, respectively. Average ± s.d. of results from four independent experiments are shown. Levels of expression of selected ToxR(GpA)MBP constructs as analyzed by immunoblotting are shown at the bottom.

Finally, the effect of ionizable residues in TM packing in bacterial cytoplasmic membranes was assessed using the ToxCAT assay [33]. This assay uses a chimeric construct composed of the ToxR N-terminal transcriptional activation domain [34] fused to the GpA TM segment and a C-terminal maltose binding protein (MBP) domain (Fig. 5A). TM-mediated dimerization of the chimera in the *E. coli* inner membrane results in transcriptional activation of a reporter gene encoding chloramphenicol acetyltransferase (CAT), with the level of CAT protein expression indicating the strength/intensity of TM helix-helix interactions. After transformation of these ToxCAT constructs into *E. coli* NT326 cells, we tested the ability of the wild-type and mutant fusion proteins carrying ionizable residues to complement the *malE* phenotype of the NT326 strain by growing each construct on plates containing maltose as the sole carbon source. Cells containing a construct that lack a TM segment do not grow (pccKAN), but the wild-type and all point mutants support growth on maltose (Fig. 5B), indicating that the MBP domains of these chimeric proteins are properly targeted to the periplasm of the NT326 cells. Consequently, the expected topology (Fig. 5A) is being achieved by these proteins, in agreement with GpA wild-type and point mutants in ToxR [35] and ToxCAT [33] assays. Dimerization of wild-type and mutant sequences carrying ionizable residues was assessed along with a GpA point mutant (G83I) that disrupts homodimerization as negative control. The I85D mutant was found to dimerize in this system to about 35% of the level shown by wild-type GpA (Fig. 5C). Interestingly, L89D mutant, which precludes dimer formation in the presence of SDS micelles (Fig. 3C), appears to retain some dimerization capacity (21±4%, normalized dimerization), which highlights the influence of the specific lipid environment during the assembly of TM segments [36]. Nevertheless, differences in TM segment length and flanking residues sequences (see Fig. S1) may alter the dimerization process in the two systems, which are difficult to rationalize. Mutation of Leu89 to lysine (L89K) had a smaller effect on TM dimerization, and double mutant I85D/L89K still retained some dimerization capacity (Fig. 5C). In agreement with these data, recent molecular dynamics simulations suggested that a lysine residue outside the contact interface could exert a significant influence on TM helix association affinity of the bacteriophage M13 major coat protein because the extent of their burial in the membrane could be different in monomers and dimers [37]. Together, our data indicate that the presence of ionizable residues does not preclude membrane insertion and allows dimer formation in bacterial cells.

## Conclusions

Ionizable amino acid residues are functionally and/or structurally important residues in membrane proteins. Therefore, although the insertion of such residues into the membrane hydrophobic core may be energetically unfavourable, there is often a functional and/or structural necessity to accommodate them. In the light of our experiments it can be concluded that nonpolar to ionizable point substitutions at specific positions away from the dimer interface (‘lipid facing’) in combination with a N-terminal GxxxG motif does not preclude neither the dimerization process nor TM helix insertion, while point mutations of nonpolar (or polar nonionizable) to ionizable residues in the ‘helix facing’, *e.g.* I91D/E, I91K/R, or T87D strongly compromise dimer formation. These notions need to be considered if we are to develop a predictive understanding of TM helix interactions in membrane proteins.

## Materials and Methods

### Helix data set

All α-helical membrane proteins deposited in the MPTOPO database (last updated on January 19^th^, 2010) [20], and thus with known membrane insertion topology, were selected. The initial set was further filtered by: (i) removing any entry of unknown structure as based on the MPTOPO entry classification (*i.e.*, keeping only entries described as “3D_helix” and “1D_helix”); and (ii) removing redundant pairs at 80% sequence identity by applying the *cd-hit* program [38]. The final data set of TM helices contained 170 non-redundant structures, 837 TM helices, and 20,079 amino acids. Furthermore, to properly analyze the amino acid propensities in single membrane spanning TM helices, we discarded any helix shorter than 17 amino acids or larger than 38 amino acids. The resulting TM data subset contained 792 TM helices, and 19,356 amino acids.

### Plasmid constructs

Construction of the plasmids encoding the His-tagged chimeric proteins (SN/GpA) have been described [13], [39]. Mutations at the TM fragment of GpA were obtained by site-directed mutagenesis using the QuikChange site directed mutagenesis kit (Stratagene, La Jolla, California). Introduction of the TM segment from GpA into the Lep sequence was described elsewhere [16]. The ToxCAT vector pccKAN, and the derivatives carrying the TM domain of GpA (pccGpA) and a disruptive GpA mutant (pccGpA-G83I) fused to the ToxR transcription activator and to maltose-binding protein (MBP) were described previously [33]. All mutants were confirmed by DNA sequencing.

### Protein expression and purification

Overexpression and purification of His-tagged SN/GpA constructs from transformed *Escherichia coli* BL21 (DE3) cells was performed as described [40]. *In vitro* transcription/translation of Lep-derived constructs was done in the presence of reticulyte lysate and [^35^S]-labeled amino acids as described [16].

### SDS-PAGE analysis

Purified SN/GpA proteins were loaded onto SDS 12% polyacrylamide mini-gels. The loading buffer contained 2% (w/v) SDS, and samples were boiled for five minutes prior to electrophoresis. Gels were stained with Coomassie blue, and the percentages of monomer and dimer were estimated with a ImageQuantTM LAS 4000mini Biomolecular Imager (GE Healthcare). Gels with radioactive Lep-derived samples were dried at 80°C and scanned using a Fuji FLA-3000 phosphorimager using the ImageGauge software.

### ToxCAT methods

Plasmids encoding ToxR(GpA)MBP chimerae were transformed into *Escherichia coli* NT326 cells (kindly provided by D. M. Engelman) and plated onto Luria Bertani (LB) plates (with 50 µg/ml ampicillin, 25 µg/ml streptomycin); colonies were inoculated into LB medium (with 50 µg/ml ampicillin, 25 µg/ml streptomycin), and glycerol stocks were made at A_600_≈0.2 and stored at −80°C. LB cultures (with 50 µg/ml ampicillin, 25 µg/ml streptomycin) were inoculated from frozen glycerol stocks and grown at 37°C until approximately A_420_≈0.6, when culture densities were equalized by dilution into fresh culture tubes, and 6.0 A_420_ units of cells were harvested by centrifugation and washed with 0.4 ml of sonication buffer (25 mM Tris-HCl, 2 mM EDTA, pH 8.0) [41]. Cells were then resuspended in 0.6 ml of sonication buffer and lysed by probe sonication. After removing an aliquot (20 µl) for Western blot analysis, the remaining lysate was clarified by centrifugation at 13,000×g, and the supernatant was stored on ice until the spectrophotometric assay was performed. All constructs conferred the ability to grow on maltose plates to the *malE^–^* strain NT326, which indicates that proper membrane insertion of the ToxR(GpA)MBP fusion protein has occurred [33]. For maltose complementation assays, *E. coli* NT326 cells expressing ToxR(GpA)MBP constructs were streaked on M9 minimal media plates containing 0.4% maltose as the only carbon source, and incubated for 3 days at 37°C. All constructs showed similar expression levels of ToxR(GpA)MBP fusion protein as determined by Western blot using an anti-MBP antibody. The self-association ability of the TM domain triggers expression of a chloramphenicol transferase (*cat*) gene reporter and production of CAT protein can be quantified by a CAT-ELISA kit (Roche Diagnostics) [42]. CAT measurements and construct expression measurements were performed in at least triplicate and were normalized for the relative expression level of each construct using Western blotting [43]. All constructs showed similar expression levels of ToxR(GpA)MBP fusion proteins as determined by Western blot using an anti-MBP antibody. For Western blots samples were mixed with equal volumes of 2× SDS-PAGE sample buffer heated to 95°C for 10 min, separated on 10% (w/v) polyacrylamide mini-gels, blotted onto nitrocellulose membranes, and blocked in skim milk. ToxR(GpA)MBP chimera were detected with biotinylated anti-MBP primary antibody (NEB) and visualized with streptavidin-horseradish peroxidase conjugate and ECL reagent (GE Healthcare). Bands were quantified with an ImageQuantTM LAS 4000mini Biomolecular Imager (GE Healthcare).

## Supporting Information

Figure S1 **TM segments and flanking residues sequences.** The primary sequences of the GpA TM regions used in both the SDS-PAGE and ToxCAT analyses are shown. Hydrophobic residues are boxed in yellow and flanking residues are highlighted (italic).
(EPS)
